# Influence of Design Parameters on Mechanical Behavior of Multi-Bolt, Countersunk C/SiC Composite Joint Structure

**DOI:** 10.3390/ma16196352

**Published:** 2023-09-22

**Authors:** Hongcui Wang, Lijia Guo, Weijie Li, Mengshan Zhang, Yiqiang Hong, Wei Yang, Zhongwei Zhang

**Affiliations:** 1 School of Civil Engineering, Beijing Jiaotong University, Beijing 100044, China; 2 Beijing Mechanical Equipment Institute, Beijing 100039, China; 3 Beijing System Design Institute of Electro-Mechanic Engineering, Beijing 100854, Chinayangwei_vip@hotmail.com (W.Y.); 4 Institute of Advanced Structure Technology, Beijing Institute of Technology, Beijing 100081, China

**Keywords:** multi-bolt, countersunk C/SiC composite joint structures, 3D-Hashin progressive damage model, peak load, weight increment efficiency, bolt load distribution

## Abstract

Aerospace vehicle connection constructions are in urgent need of joint structures with excellent aerodynamic profiles and environmental adaptability. To address issues such as poor aerodynamic profile, material thermal expansion coefficient mismatch, and limited joint structure evaluation indexes, a multi-bolt, countersunk C/SiC composite joint structure is presented in this study. The development of a 3D Hashin progressive damage model and its dedicated solver code is presented. The validity of the model is confirmed by comparing simulation results with experimental data. Three evaluation indexes are proposed, peak load, weight increment efficiency, and bolt load distribution, to thoroughly evaluate the mechanical performance of multi-bolt, countersunk C/SiC composite joint structures. Using the proposed model and evaluation indices, we evaluate sixteen different designs of multi-bolt, countersunk C/SiC composite joint structures and analyze how design parameters affect their mechanical properties and damage patterns. The results show that the best mechanical properties of the joint structure are achieved when the ratio of bolt pitch to through hole diameter is 3, the ratio of bolt spacing between columns to through hole diameter is 4, the ratio of the distance between the free edge of the substrate to through hole diameter is 1.5, the ratio of through hole diameter to specimen thickness is 1.7, and the ratio of the distance between the edge of the substrate to through hole diameter is 1.5.

## 1. Introduction

Composite bolted structures are extensively applied in the joint structure of aerospace vehicles due to their high load-bearing capacity, superior reliability, and ability to be assembled and disassembled repeatedly [1,2,3]. As the load-carrying capacity of aerospace vehicles increases and their service environment becomes more complex [4,5,6], higher demands are imposed on their joint structure. On the one hand, aerospace vehicles in a thermo-mechanical-oxygenic coupling environment require a good aerodynamic profile of the joint structure [7,8]. On the other hand, matching the coefficient of thermal expansion for the materials of the joint structure is required to avoid thermal stresses and thermal deformations, and high-temperature resistance is also required [9,10,11]. Countersunk C/SiC composite joint structures can meet these requirements, so there is an urgent need to carry out research on their design and performance evaluation.

In terms of manufacture, single-lap or double-lap joint structure with a single bolt has been studied in depth [12,13,14,15,16,17]. Tang et al. [12] found that the dominant failure modes for both single-lap and double-lap joint structures were bearing failure and net-tension. Zhou et al. [13] investigated the effect of the pin profiles on the stress concentration and load-carry capacity of the pin-loaded single-lap joints subjected to tensile loading. In actual aerospace vehicles, however, the joint structure is often a combination of multiple bolts, which bear the load together [18,19,20,21,22]. Mandal et al. [18] proposed a hybrid approach for global sensitivity analysis of FRP composite multi-bolt joints. Taheri-Behrooz et al. [21] presented an analytical approach to determine load distribution in single-column multi-bolt composite joints by taking into account the effect of material nonlinearity of the joint members. In terms of performance evaluation, failure patterns of joint structures are of interest to many researchers. For example, progressive damage models with different failure criteria and material degradation rules were used to predict the failure patterns of carbon fiber reinforced polymer (CFRP) composites [20,23]. The characteristic curve method and the failure envelope method were used to predict the failure of joint structures [24]. In addition, the load-bearing capacity was often taken to characterize the performance of a structure [25,26]. For example, Ding et al. [27] evaluated the mechanical properties of lap joints in CFRP plates in terms of load-carrying capacity and joint efficiency. Zhou et al. [28] evaluated the performance of bolted joints in terms of degradation after reaching load-carrying capacity. However, the single indicator (i.e., load-bearing capacity) can hardly provide a comprehensive assessment of the composite material. Consequently, there is an imperative need to develop a reasonable and comprehensive evaluation method for the design of joint structures.

In summary, the joint structure currently suffers from a variety of problems. The thermal expansion coefficient of the material is not matched, the structure shape is not smooth, the practical application is often multi-bolt rather than single-bolt, and there is a limitation of the current evaluation method for the design of the joint structure. In order to solve the above problems, in this study, a multi-bolt, countersunk C/SiC composite joint structure was designed, and an evaluation method for its design strategy was proposed. A 3D-Hashin progressive damage model for predicting the mechanical properties of C/SiC composite was developed and validated. Sixteen sets of joint parameters were designed. Their mechanical performance and failure modes were obtained and analyzed by the validated model with simulation. Three evaluation indices, i.e., peak loads, weight increment efficiency, and bolt load distribution, were proposed to comprehensively evaluate the design of the multi-bolt, countersunk C/SiC composite joint structure.

## 2. Models

### 2.1. Geometric Model

**Figure 1:**
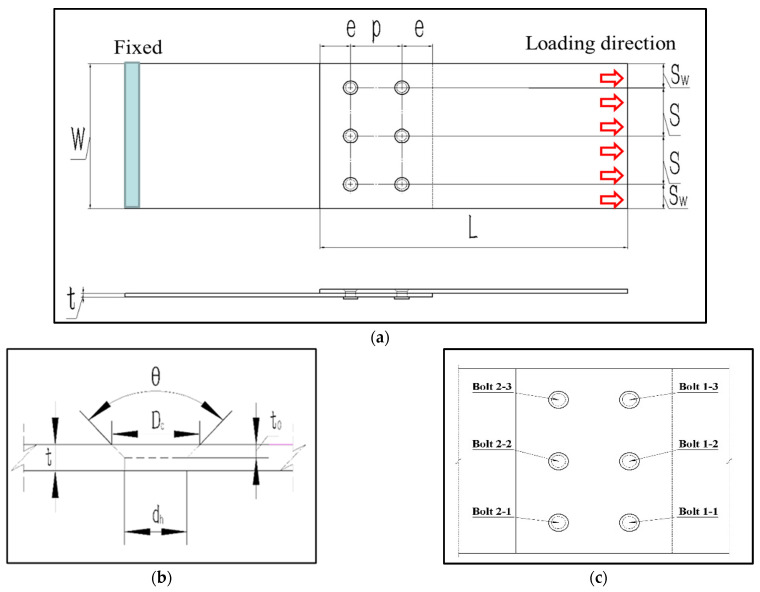
Geometric models for (**a**) Multi-bolt and countersunk C/SiC composite joints head bolts; (**b**) Countersunk head bolts; (**c**) Location and labeling of the individual bolts.

The geometry of the joint is based on the ASTM standard D5961/D 5961M-10 and GB 5279-85, as illustrated in Figure 1a [29,30]. In order to determine the mechanical properties and bolt load distribution of the multi-bolted joint structure and to provide useful data for the design of joints, the multi-bolt (two rows and three columns) joint structures were designed. Two composite rectangular substrate plates with length *L*, width *W*, and a through hole diameter *d_h_* are considered. Here, *e* is the distance between the free edge of the substrate and the center of its hole, *p* is the bolt pitch between rows of the multi-bolted joint specimens, *S_w_* is the distance between the upper or the lower edge of the substrate and the center of the holes near edges, *S* is the bolt spacing between columns of the multi-bolted joint specimens, *t* is the thickness of specimens. As shown in Figure 1b, *θ* is the angle of the countersunk, *D_c_* is the bolt-head diameter, and *t_0_* is the countersunk height. The blue area in Figure 1a is the fixed edge, and the direction of the red arrow is the loading direction. For the multi-bolt, countersunk C/SiC composite joints, the distribution of countersunk bolts with labels is shown in Figure 1c.

### 2.2. Mathematical Model

Based on the 3D-Hashin progressive damage model [20], the failure modes associated with fiber tensile failure, fiber compression failure, matrix tensile failure, and matrix compression failure can be obtained. The constitutive equation for the composite is given as:

(1) σ=Cε

where ***σ*** is the stress, ***ε*** is the strain, and ***C*** is the initial stiffness components of the element. Equation (1) can be represented more explicitly as follows:

(2) $$\left[\begin{array}{c} \sigma_{11}\\ \sigma_{22}\\ \sigma_{33}\\ \tau_{12}\\ \tau_{13}\\ \tau_{23} \end{array}\right]=\left[\begin{array}{cccccc} C_{11} & C_{12} & C_{13} & 0 & 0 & 0\\ C_{21} & C_{22} & C_{23} & 0 & 0 & 0\\ C_{31} & C_{32} & C_{33} & 0 & 0 & 0\\ 0 & 0 & 0 & C_{44} & 0 & 0\\ 0 & 0 & 0 & 0 & C_{55} & 0\\ 0 & 0 & 0 & 0 & 0 & C_{66} \end{array}\right]\left[\begin{array}{c} \varepsilon_{11}\\ \varepsilon_{22}\\ \varepsilon_{33}\\ \gamma_{12}\\ \gamma_{13}\\ \gamma_{23} \end{array}\right]$$

where

(3) $$\begin{array}{l} C_{11}=\frac{1-\nu_{23}\nu_{32}}{E_{22}E_{33}\Delta },C_{12}=\frac{\nu_{21}+\nu_{23}\nu_{31}}{E_{11}E_{33}\Delta },C_{13}=\frac{\nu_{31}+\nu_{21}\nu_{32}}{E_{11}E_{22}\Delta },\\ C_{21}=C_{12},C_{22}=\frac{1-\nu_{13}\nu_{31}}{E_{11}E_{33}\Delta },C_{23}=\frac{\nu_{32}+\nu_{12}\nu_{31}}{E_{11}E_{22}\Delta },\\ C_{31}=C_{13},C_{32}=C_{23},C_{33}=\frac{1-\nu_{12}\nu_{21}}{E_{11}E_{22}\Delta },\\ C_{44}=G_{12},C_{55}=G_{13},C_{66}=G_{23},\\ \Delta =\frac{1-\nu_{12}\nu_{21}-\nu_{23}\nu_{32}-\nu_{13}\nu_{31}-2\nu_{12}\nu_{23}\nu_{31}}{E_{11}E_{22}E_{33}} \end{array}$$

where *ν_ij_* (*I, j* = 1, 2, 3) are undamaged material Poisson’s ratios, *E_ij_* (*I, j* = 1, 2, 3; *I* = *j*), *G*_12_, *G*_13_ and *G*_23_ are undamaged material moduli.

To predict the initial damage in composites, the initiation failure criteria can be expressed as follows [20]:

Warp fiber tensile failure ($\sigma_{11}\geq 0$),

(4) $${\left(f_{X}^{T}\right)}^{2}={\left(\frac{\sigma_{11}}{X_{T}}\right)}^{2}\geq 1$$

Warp fiber compression failure ($\sigma_{11}<0$),

(5) $${\left(f_{X}^{C}\right)}^{2}={\left(\frac{\sigma_{11}}{X_{C}}\right)}^{2}\geq 1$$

Weft fiber tensile failure ($\sigma_{22}\geq 0$),

(6) $${\left(f_{Y}^{T}\right)}^{2}={\left(\frac{\sigma_{22}}{Y_{T}}\right)}^{2}\geq 1$$

Weft fiber compression failure ($\sigma_{22}<0$),

(7) $${\left(f_{Y}^{C}\right)}^{2}={\left(\frac{\sigma_{22}}{Y_{C}}\right)}^{2}\geq 1$$

Matrix tension failure ($\sigma_{33}\geq 0$),

(8) $${\left(f_{Z}^{T}\right)}^{2}={\left(\frac{\sigma_{33}}{Z_{T}}\right)}^{2}+{\left(\frac{\tau_{13}}{S_{13}}\right)}^{2}+{\left(\frac{\tau_{23}}{S_{23}}\right)}^{2}\geq 1$$

Matrix compression failure ($\sigma_{33}<0$),

(9) $${\left(f_{Z}^{C}\right)}^{2}={\left(\frac{\sigma_{33}}{Z_{C}}\right)}^{2}+{\left(\frac{\tau_{13}}{S_{13}}\right)}^{2}+{\left(\frac{\tau_{23}}{S_{23}}\right)}^{2}\geq 1$$

where *X*, *Y,* and *Z* represent strengths in the *x*, *y*, and *z* direction of the composite, respectively, and subscripts *T* and *C* mean the tension and compression. *S_ij_* (*I*, *j* = 1, 2, 3) represents the shear strain strength in the *ij*-plane. $f_{j}^{i}$ (*I* = *T*, *C*; *j* = *X*, *Y*, *Z*) are the loading function, and once the value of any indicator is equal or exceeds 1, the corresponding failure appears.

By utilizing the stress-strain relationship presented in Equation (10) and based on the initial stress-based 3D-Hashin progressive damage model failure criterion [31], we adapted it to a strain-based 3D-Hashin progressive damage model failure criterion:

(10) $$\sigma_{11}=E_{11}\varepsilon_{11},\;\sigma_{22}=E_{22}\varepsilon_{22},\;\sigma_{33}=E_{33}\varepsilon_{33},\;\tau_{13}=G_{13}\gamma_{13},\;\tau_{23}=G_{23}\gamma_{23},\;\;X_{T}=E_{11}X_{T}^{\varepsilon },\;X_{C}=E_{11}X_{C}^{\varepsilon },\;Y_{T}=E_{22}Y_{T}^{\varepsilon },\;Y_{C}=E_{22}Y_{C}^{\varepsilon },\;Z_{T}=E_{33}Z_{T}^{\varepsilon },\;\;Z_{C}=E_{33}Z_{c}^{\varepsilon },\;S_{13}=G_{13}S_{13}^{\varepsilon },\;S_{23}=G_{23}S_{23}^{\varepsilon }$$

where $X_{T}^{\varepsilon }$, $X_{C}^{\varepsilon }$, $Y_{T}^{\varepsilon }$, $Y_{C}^{\varepsilon }$, $Z_{T}^{\varepsilon }$ and $Z_{c}^{\varepsilon }$ represent strain strengths in *x*, *y*, and *z* direction of composite, and subscripts *T* and *C* mean the tension and compression. $S_{13}^{\varepsilon }$ and $S_{23}^{\varepsilon }$ signifies the corresponding shear strain strengths.

The strain-based 3D-Hashin progressive damage model failure criterion is expressed as:

Warp fiber tensile failure ($\varepsilon_{11}\geq 0$),

(11) $${\left(f_{X}^{T}\right)}^{2}={\left(\frac{\varepsilon_{11}}{X_{T}^{\varepsilon }}\right)}^{2}\geq 1$$

Warp fiber compression failure ($\varepsilon_{11}<0$),

(12) $${\left(f_{X}^{C}\right)}^{2}={\left(\frac{\varepsilon_{11}}{X_{C}^{\varepsilon }}\right)}^{2}\geq 1$$

Weft fiber tensile failure ($\varepsilon_{22}\geq 0$),

(13) $${\left(f_{Y}^{T}\right)}^{2}={\left(\frac{\varepsilon_{22}}{Y_{T}^{\varepsilon }}\right)}^{2}\geq 1$$

Weft fiber compression failure ($\varepsilon_{22}<0$),

(14) $${\left(f_{Y}^{C}\right)}^{2}={\left(\frac{\varepsilon_{22}}{Y_{C}^{\varepsilon }}\right)}^{2}\geq 1$$

Matrix tension failure ($\varepsilon_{33}\geq 0$),

(15) $${\left(f_{Z}^{T}\right)}^{2}={\left(\frac{\varepsilon_{33}}{Z_{T}^{\varepsilon }}\right)}^{2}+{\left(\frac{\gamma_{13}}{S_{13}^{\varepsilon }}\right)}^{2}+{\left(\frac{\gamma_{23}}{S_{23}^{\varepsilon }}\right)}^{2}\geq 1$$

Matrix compression failure ($\varepsilon_{33}<0$),

(16) $${\left(f_{Z}^{C}\right)}^{2}={\left(\frac{\varepsilon_{33}}{Z_{C}^{\varepsilon }}\right)}^{2}+{\left(\frac{\gamma_{13}}{S_{13}^{\varepsilon }}\right)}^{2}+{\left(\frac{\gamma_{23}}{S_{23}^{\varepsilon }}\right)}^{2}\geq 1$$

Once damage has been initiated, the corresponding damage variable for a specific damage criterion is [32]:

(17) $$d_{X}^{T}=1-\frac{1}{f_{X}^{T}}e^{\left[C_{11}{\left(X_{T}^{\varepsilon }\right)}^{2}\left(1-f_{X}^{T}\right)L^{c}/G_{f}\right]}$$

(18) $$d_{X}^{C}=1-\frac{1}{f_{X}^{C}}e^{\left[C_{11}{\left(X_{C}^{\varepsilon }\right)}^{2}\left(1-f_{X}^{C}\right)L^{c}/G_{f}\right]}$$

(19) $$d_{Y}^{T}=1-\frac{1}{f_{Y}^{T}}e^{\left[C_{22}{\left(Y_{T}^{\varepsilon }\right)}^{2}\left(1-f_{Y}^{T}\right)L^{c}/G_{f}\right]}$$

(20) $$d_{Y}^{C}=1-\frac{1}{f_{Y}^{C}}e^{\left[C_{22}{\left(Y_{C}^{\varepsilon }\right)}^{2}\left(1-f_{Y}^{C}\right)L^{c}/G_{f}\right]}$$

(21) $$d_{Z}^{T}=1-\frac{1}{f_{Z}^{T}}e^{\left[C_{33}{\left(Z_{T}^{\varepsilon }\right)}^{2}\left(1-f_{Z}^{T}\right)L^{c}/G_{m}\right]}$$

(22) $$d_{Z}^{C}=1-\frac{1}{f_{Z}^{C}}e^{\left[C_{33}{\left(Z_{C}^{\varepsilon }\right)}^{2}\left(1-f_{Z}^{C}\right)L^{c}/G_{m}\right]}$$

where *L^c^* is the characteristic length associated with the material point. *G_f_* is the fracture energy of the fiber, *G_m_* is the fracture energy of the matrix. $d_{i}^{j}$ (*I* = *X*, *Y*, *Z*; *j* = *T*, *C*) represent the damage status of the element, which ranges from 0 to 1, with the value increasing to indicate the degree of damage, with 0 indicating no damage to the element and 1 indicating complete damage to the element.

Warp fiber, weft fiber, and matrix have different damage variables during tension and compression, which will be denoted by $d_{f}^{X}$, $d_{f}^{Y}$ and $d_{m}^{Z}$ as follows:

(23) $$d_{f}^{x}=\max(d_{X}^{T},d_{X}^{C}),\;d_{f}^{y}=\max(d_{Y}^{T},d_{Y}^{C}),\;d_{m}^{z}=\max(d_{Z}^{T},d_{Z}^{C})$$

Degradation and failure of the material occur after damage has occurred and follow the damage evolution law described above. The constitutive equation characterizing the damage evolution can be expressed as:

(24) $$\sigma =C^{d}\varepsilon$$

where *C^d^* represents the damage stiffness matrix containing the material elastic coefficients and damage variables. The expansion constitutive equation of Equation (24) is available in literature [33]:

(25) $$C^{d}=\left[\begin{array}{cccccc} {\left(1-d_{f}^{x}\right)}^{2}C_{11} & \left(1-d_{f}^{x}\right)\left(1-d_{f}^{y}\right)C_{12} & \left(1-d_{f}^{x}\right)\left(1-d_{m}^{z}\right)C_{13} & 0 & 0 & 0\\ & {\left(1-d_{f}^{y}\right)}^{2}C_{22} & \left(1-d_{f}^{y}\right)\left(1-d_{m}^{z}\right)C_{23} & 0 & 0 & 0\\ & & {\left(1-d_{m}^{z}\right)}^{2}C_{33} & 0 & 0 & 0\\ & & & \left(1-d_{f}^{x}\right)\left(1-d_{f}^{y}\right)C_{44} & 0 & 0\\ & symmetric & & & \left(1-d_{f}^{x}\right)\left(1-d_{m}^{z}\right)C_{55} & 0\\ & & & & & \left(1-d_{f}^{y}\right)\left(1-d_{m}^{z}\right)C_{66} \end{array}\right]$$

Using the damage criterion of the 3D-Hashin progressive damage in the form of strain, the Jacobian matrix of fibers and yarns can be expressed as follows:

(26) $$\begin{array}{ll} \frac{\partial \sigma }{\partial \varepsilon } & =C^{d}+\frac{\partial C^{d}}{\partial \varepsilon }:\varepsilon \\ & =C^{d}+\left(\frac{\partial C^{d}}{\partial d_{f}^{x}}:\varepsilon \right)\left(\frac{\partial d_{f}^{x}}{\partial f_{x}}\frac{\partial f_{x}^{}}{\partial \varepsilon }\right)\\ & +\left(\frac{\partial C^{d}}{\partial d_{f}^{y}}:\varepsilon \right)\left(\frac{\partial d_{f}^{y}}{\partial f_{y}}\frac{\partial f_{y}}{\partial \varepsilon }\right)\\ & +\left(\frac{\partial C^{d}}{\partial d_{m}^{z}}:\varepsilon \right)\left(\frac{\partial d_{m}^{z}}{\partial f_{z}}\frac{\partial f_{z}}{\partial \varepsilon }\right) \end{array}$$

where *f_x_*, *f_y_*, and *f_z_* are the load functions corresponding to $d_{f}^{X}$, $d_{f}^{Y}$, and $d_{m}^{Z}$.

The weight increment efficiency *η* of a composite joint is defined as the breaking load *P* of the joint divided by the weight increment Δ*G* in the joint area and can be calculated according to the following Equation [34]:

(27) η=P/ΔG

where *P* is the breaking load of the connection, and Δ*G* is the weight increment in the connection area.

**Figure 2:**
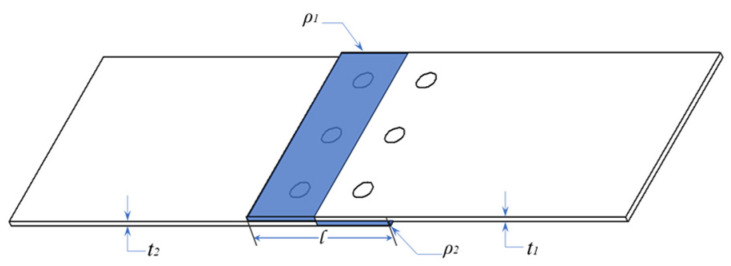
Calculation of single-lap weight increments and joint efficiency.

The weight increment is relative to the absence of the joints. For a single-lap joint, the part of the shaded area in Figure 2 is the weight increment part. The calculation formula is:

(28) $$\Delta G=l\left(\rho_{1}t_{1}+\rho_{1}t_{2}\right)W$$

where *l* is the length of the overlapping part of the connection area; *ρ*_1_, *ρ*_2_ are the densities of the upper and lower substrates, respectively. 

In terms of material efficiency, the higher the weight increment efficiency of the composite, the better. The higher the weight increment efficiency, the higher the load-bearing capacity of the joint structure for the same amount of material.

## 3. Validation

### 3.1. Material Parameters

In this paper, C/SiC composites are used for both the bolts and the substrate plates, and the material properties are shown in Table 1 [20].

### 3.2. Simulation and Experimental Results

To carry out a non-linear progressive failure analysis of C/SiC composite structures, ABAQUS (Abaqus 2021 version) finite element software was used. Meanwhile, we embedded a user-defined subroutine UMAT into ABAQUS, including the relevant constitutive model, initiation failure criteria, and damage criterion based on the 3D-Hashin progressive damage model. The C/SiC composite joint structure is modeled using solid cells and using the cell type C3D8R.

**Figure 3:**
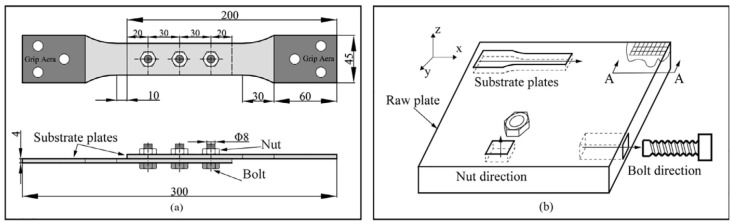
An all-C/SiC single lap, three-bolt composite joint: (**a**) Configuration and geometry; (**b**) Directions of composite substrate plates, bolts, and nuts [20].

Two all-C/SiC composite single-lap three-bolt joints were investigated in this study [20]. Figure 3a presents the configuration, geometry, and fabrication details of the specimens, which had their substrates and C/SiC bolts/nuts cut, machined, and then assembled from raw plates. As depicted in Figure 3b, the axes of the substrates and bolts were aligned longitudinally along the *x*-direction, whereas the nut’s axis lay perpendicular to the joint plane along the *z*-direction.

**Figure 4:**
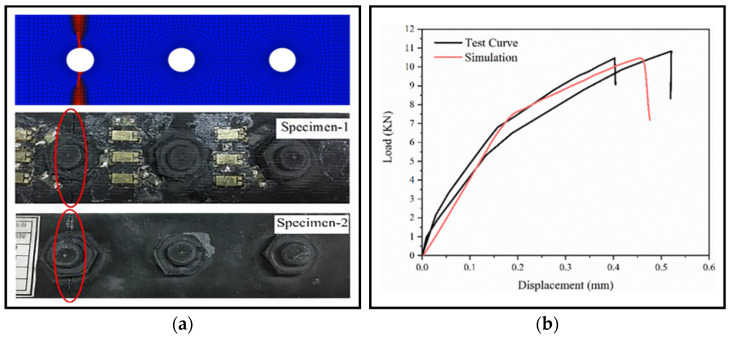
Comparison of numerical simulation and experimental results: (**a**) Failure mode; (**b**) Load-displacement curves.

The simulation results were compared with experimental results from the literature. The final failure modes and force-displacement curves of the simulation and experiment were compared, as shown in Figure 4. From the failure mode diagram, it can be seen that the simulation results have the same material pattern as the experimental results, both due to tensile failure of the upper lap plate at the bolted joint. From the load-displacement curves of the bolted joint structure, it can be seen that the peak load obtained experimentally is 10.819 kN versus 10.498 kN. The peak load obtained from the finite element simulation of the joint structure is 10.453 kN. The relative error between the results obtained by the finite element simulation and the average of the experimental results is less than 5%, which proves that the finite element analysis procedure and the UMAT subroutine are appropriate.

## 4. Results and Discussion

In this paper, 16 different multi-bolt, countersunk C/SiC composite joint structures were designed with the following design parameters: *P/d_h_*, *S/d_h_*, *e/d_h_*, *d_h_/t*, *S_w_/d_h_*, as shown in Table 2. The through hole diameters are all set to 10 mm in all designs.

### 4.1. Effect of P/d_h_ on Mechanical Behavior of Joint Structure

**Figure 5:**
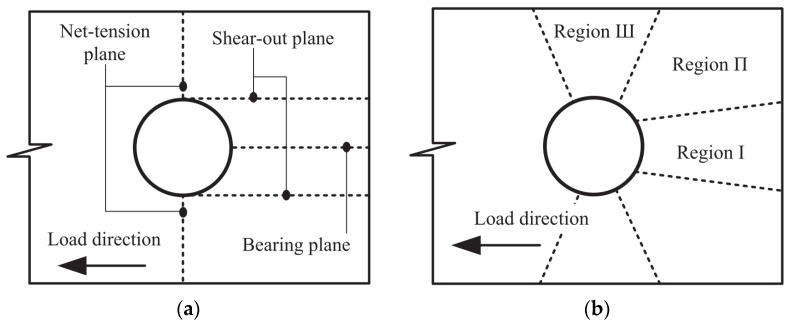
Damage mechanism identification around the contact zone [35]. (**a**) Definition of failure planes; (**b**) Definition of damage regions.

To better illustrate the failure mode, the failure plane is defined as shown in Figure 5a [35]. Based on the failure plane, the model shown in Figure 5b is divided into three separate regions: (1) Bearing damage region I, (2) Shear-out damage region II, and (3) Net-tension damage region III.

**Figure 6:**
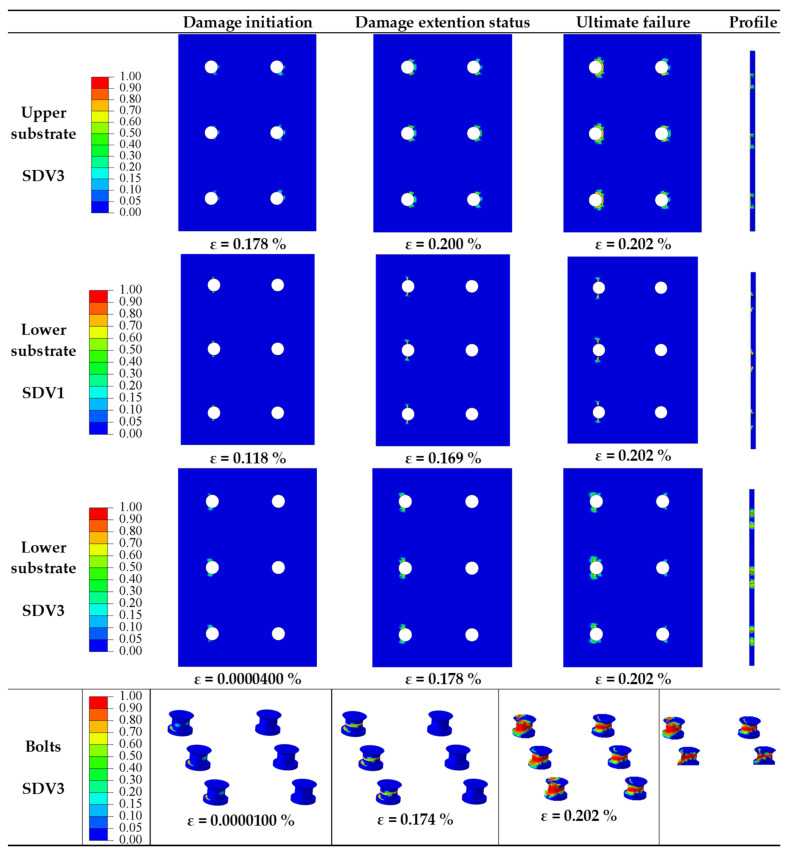
Damage processes in joint structures at *P/d_h_* = 5.

The objective of this section is to investigate the impact of *P/d_h_* on the mechanical behavior of multi-bolt, countersunk C/SiC composite joint structures. When *S/d_h_* = 5, *e/d_h_* = 3, *d_h_/t* = 2.5, *S_w_/d_h_* = 2.5, and the preload force is 2 kN, the numerical model is used to calculate and analyze the effect of *P/d_h_* of 2, 3, 4, and 5, respectively, on the damage process of the joint structure. Their design strategies are numbered 1, 2, 3, and 4, respectively. Analysis and comparison showed that the damage process and damage patterns were the same for *P/d_h_* of 2, 3, 4, and 5, so the damage process for *P/d_h_* = 5 was taken for analysis. The damage to the joint structure is more obvious around the bolt and bolt hole, so the part near the bolt hole is taken for damage analysis; the damage process is shown in Figure 6.

It is clear from the diagram that the matrix damage (SDV3) occurred to the upper substrate. The damage starts in the bearing damage region near the loaded side of a row of bolt holes, and then it expands in the shear-out damage region and bearing damage region. When the load reaches the peak load, the damage is produced in the two rows of bolts in the shear-out damage region and the bearing damage region. It can be seen that the type of damage to the upper substrate is the matrix compression failure caused by the combination of shear and compression stresses on the upper substrate. It is found that the warp fiber damage (SDV1) to the lower substrate starts from the net-tension damage region near the bolt holes of the fixed-end row. With the increase of applied load, the damage gradually extends in the same row, and eventually, the warp fiber damage occurs in the net-tension damage region due to the tensile stress. The matrix damage (SDV3) to the lower substrate appears In the shear-out damage region of the bolt holes near the row of fixed edges and gradually expands in the shear-out damage region. The results show that the damage is mainly due to the compression of the matrix caused by the shear stress. The matrix damage can be seen in bolts. The damage initially appears at the root of the bolt in the row near the fixed edge. As the load increases, the damage extends towards the middle bolts near the fixed edge, while it also starts to appear in the middle of the bolts near the end where the load is applied. As the load continues to increase, the damage extends to the head of the bolt. The section at the moment of peak load shows that the damage is most severe in the middle of the bolt and extends to the interior of the bolt. The damage is mainly due to matrix compression failure caused by shear stress.

**Figure 7:**
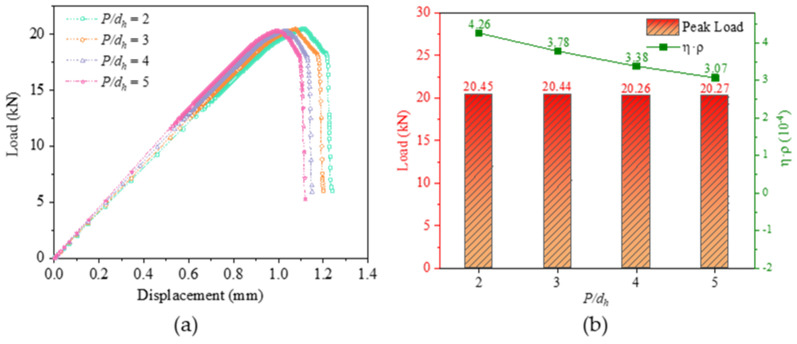
Comparison of results at different *P/d_h_*: (**a**) Load−displacement curves; (**b**) Load−bearing capacity and weight increment efficiency.

Figure 7a shows the load-displacement diagram at different *P*/*d_h_* for *S/d_h_* = 5, *e/d_h_* = 3, *d_h_/ t* = 2.5, *S_w_/d_h_* = 2.5, and preload force of 2 kN, and Figure 7b shows the peak load and weight increment efficiency at different *P/d_h_*. With the increase of *P/d_h_*, the load-bearing capacity of the structure does not change significantly, and the weight increment efficiency decreases significantly.

**Figure 8:**
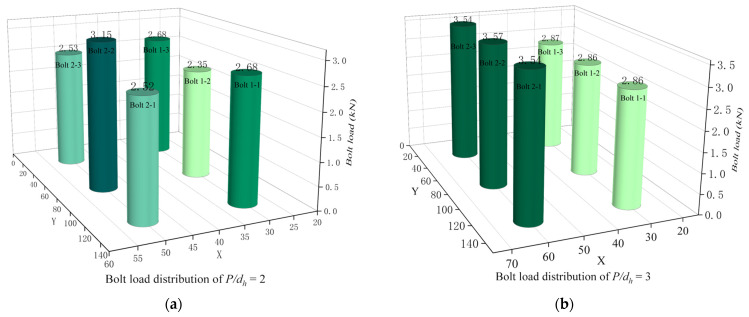

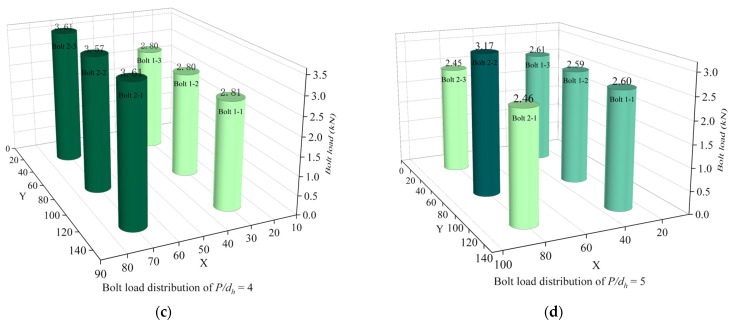
Bolt load distribution at different *P/d_h_*. (**a**) Bolt load distribution of *P/d_h_* = 2; (**b**) Bolt load distribution of *P/d_h_* = 3; (**c**) Bolt load distribution of *P/d_h_* = 4; (**d**) Bolt load distribution of *P/d_h_* = 5.

Figure 8a–d show the bolt load distribution for *P/d_h_* = 2, 3, 4, and 5, respectively. Uniform bolt load distribution results in better stability and reliability of the joint structure reduces the risk of local overload and instability of the structure, and increases the safety of the structure. A uniform distribution of bolt loads also prevents damage or fatigue fractures in certain areas due to excessive long-term stresses, thus extending the service life of the structure. At the same time, the need to strengthen certain parts of the structure due to local overloads can be avoided, thus reducing the manufacturing and maintenance costs of the structural components. As can be seen from the comparison of the four diagrams, when *P/d_h_* = 2 and *P/d_h_* = 5, the load-bearing capacity of the bolts is not uniform; when *P/d_h_* = 3 and *P/d_h_* = 4, the load-bearing capacity of each row of bolts is more uniform.

In summary, considering the effect of *P/d_h_* on the mechanical behavior of the multi-bolt, countersunk C/SiC composite joints, the load capacity of the joint structure remains basically the same as *P/d_h_* varies, but when *P/d_h_* = 3 and *P/d_h_* = 4, the bolt load is distributed more evenly for each row of bolts. In these two cases, the weight increment efficiency is greater when *P/d_h_* = 3, which allows for better material savings. Therefore, with *P/d_h_* = 3, the connection mechanism performs better with *S/d_h_* = 5, *e/d_h_* = 3, *d_h_/ t* = 2.5, *S_w_/d_h_* = 2.5, and a preload of 2 kN.

### 4.2. Effect of S/d_h_ on Mechanical Behavior of Joint Structure

**Figure 9:**
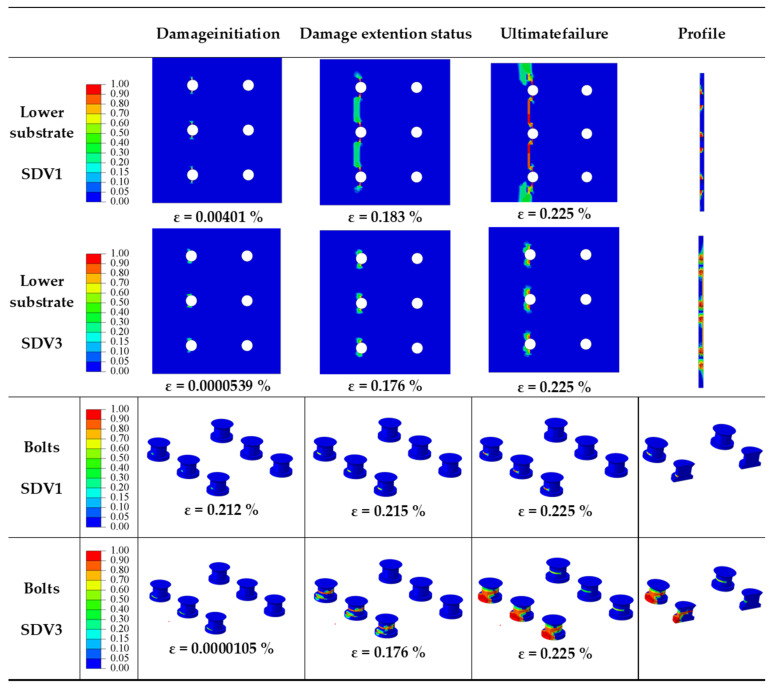
Damage processes in joint structures at *S/d_h_* = 4.

The objective of this section is to investigate the impact of *S/d_h_* on the mechanical behavior of multi-bolt, countersunk C/SiC composite joint structures when *P/d_h_* = 5, *e/d_h_* = 3, *d_h_/t* = 2.5, *S_w_/d_h_* = 2.5, and the preload force is 2 kN. The damage process and damage patterns are the same for *S/d_h_* = 2, 3, and 4, and the damage process for *S/d_h_* = 5 is shown in Figure 6. Take the damage process of *S/d_h_* = 4 for analysis; the damage process is shown in Figure 9.

The damage to the joint structure occurs at the lower substrate and at the bolts. The lower substrate showed warp fiber damage (SDV1), with the damage initially appearing in the net-tension damage region near the bolt holes in the row of bolts at the fixed end. As the load increases, the area of damage expands from the bolt holes to the area around the holes, with the damage connecting the bolt holes to each other. Finally, the area between the bolt holes is damaged in the net-tension damage region, and the damage is caused by the fibers being subjected to tensile stress. The lower substrate shows matrix damage (SDV3), which initially occurs in the shear-out damage region near the fixed end side, and as the load increases, the damage continues to expand in the shear-out damage region, followed by damage in the net-tensile region. The shear stresses caused the matrix to break in compression, and the tensile stresses also caused the matrix to break in the net-tensile region. The bolts show warp fiber damage (SDV1) and matrix damage (SDV3). The warp fiber damage initially appears at the root of the row of bolts near the fixed end, and as the load increases, the damage extends along the root. The warp fiber damage is caused by compression. The matrix damage also occurs first at the roots of the bolts in the row near the fixed end. As the load increases, the damage occurs in the middle part of the bolt, while the damage at the root of the bolt extends further. As the load increases further, the damage to the root of the bolt forms a joint area with the damage to the middle part of the bolt. As can be seen from the section, the damage to the bolt penetrates the entire section of the bolt, and the joint structure fails.

**Figure 10:**
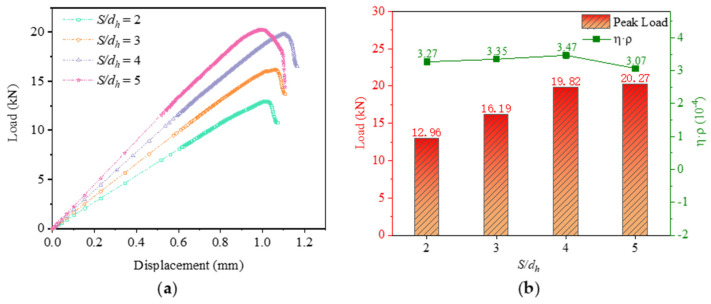
Comparison of results at different *S/d_h_*: (**a**) Load−displacement curves; (**b**) Load−bearing capacity and weight increment efficiency.

As can be seen from Figure 10a, the load-bearing capacity gradually increases as *S/d_h_* increases from 2 to 4. When *S/d_h_* increases further to 5, the load-bearing capacity of the bolt does not increase significantly. From Figure 10b, it can be seen that the weight increment efficiency shows an increasing trend during the increase of *S/d_h_* from 2 to 4 and decreases when *S/d_h_* increases to 5. This is because when *S/d_h_* = 5, the weight increment increases further with increasing column spacing, while the peak load increases less, and the weight increment efficiency decreases.

**Figure 11:**
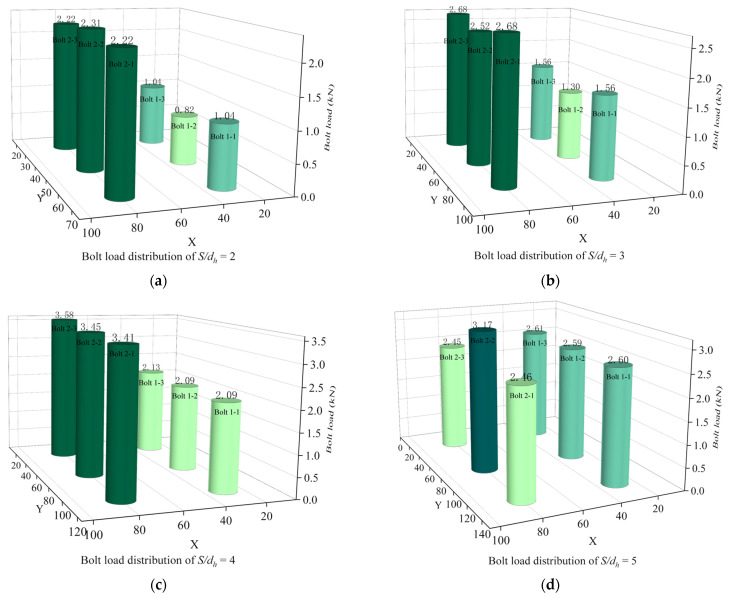
Bolt load distribution at different *S/d_h_*. (**a**) Bolt load distribution of *S/d_h_* = 2; (**b**) Bolt load distribution of *S/d_h_* = 3; (**c**) Bolt load distribution of *S/d_h_* = 4; (**d**) Bolt load distribution of *S/d_h_* = 5.

Figure 11a–d show the distribution of bolt loads for different *S/d_h_*. A comparison of the four pictures shows that the load capacity of each row of bolts is relatively uniform for *S/d_h_* = 4. The average load-bearing capacity of the two rows of bolts is not very different.

Considering the load-bearing capacity, weight increment efficiency, and bolt load distribution, the best performance of the joint structure is achieved with *S/d_h_* = 4.

### 4.3. Effect of e/d_h_ on Mechanical Behavior of Joint Structure

The objective of this section is to investigate the impact of *e/d_h_* on the mechanical behavior of multi-bolt, countersunk C/SiC composite joints. When *P/d_h_* = 5, *S/d_h_* = 5, *d_h_/t* = 2.5, *S_w_/d_h_* = 2.5, and the preload force is 2 kN, the numerical model is used to analyze the effect of *e/d_h_* of 3, 1.5, 2, and 2.5 on the damage process of the joint structure. Their connection design options are numbered 4, 8, 9, and 10. Analysis and comparison reveal a similar damage process and damage pattern for *e/d_h_* of 1.5, 2, 2.5, and 3. The damage process and damage pattern are referenced in Figure 6.

**Figure 12:**
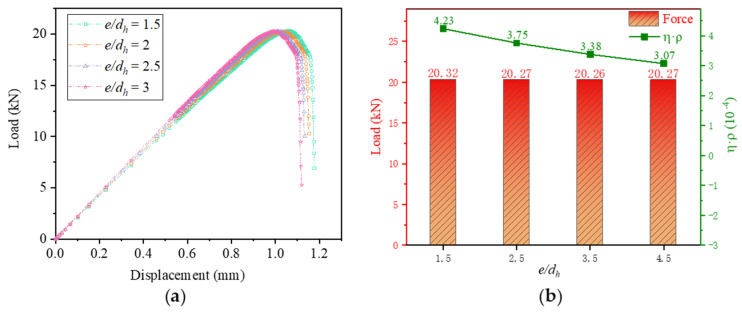
Comparison of results at different *e/d_h_*: (**a**) Load−displacement curves; (**b**) Load−bearing capacity and weight increment efficiency.

As can be seen from Figure 12a, *e/d_h_* increases from 1.5 to 3. The initial stiffness of the joint structure does not change, and the strength remains essentially unchanged. The change in *e/d_h_* has essentially no effect on the strength of the joint structure. In Figure 12b, it can be seen that the peak load does not change much, but the weight increment efficiency decreases with increasing *e/d_h_*.

**Figure 13:**
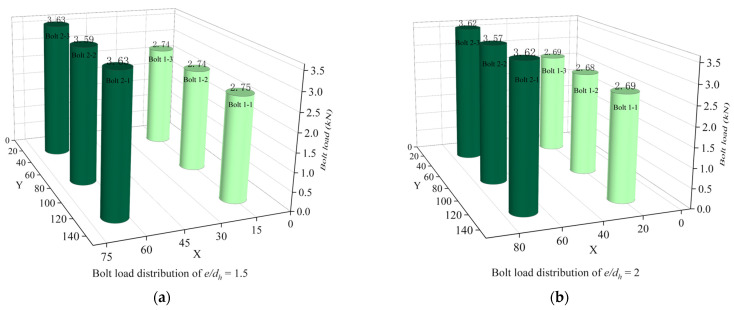

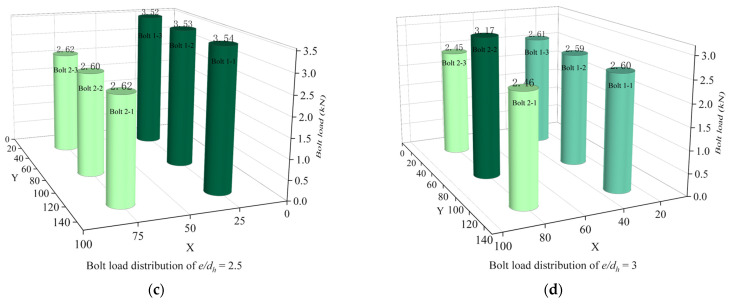
Bolt load distribution at different *e/d_h_*. (**a**) Bolt load distribution of *e/d_h_* = 1.5; (**b**) Bolt load distribution of *e/d_h_* = 2; (**c**) Bolt load distribution of *e/d_h_* = 2.5; (**d**) Bolt load distribution of *e/d_h_* = 3.

As shown in Figure 13, the bolt load distribution of *e/d_h_* in the range of 1.5 to 2.5 is relatively unified for each row of bolts. When *e/d_h_* is 3, the force on the bolts in the row near the fixed end becomes greater for the middle bolts and less for the bolts at the ends. The bolt load is not evenly distributed.

The combined load-bearing capacity, the efficiency of weight increments, and the uniformity of bolt load distribution are taken into account. The best performance of the joint structure is achieved when *e/d_h_* is 1.5.

### 4.4. Effect of d_h_/t on Mechanical Behavior of Joint Structure

**Figure 14:**
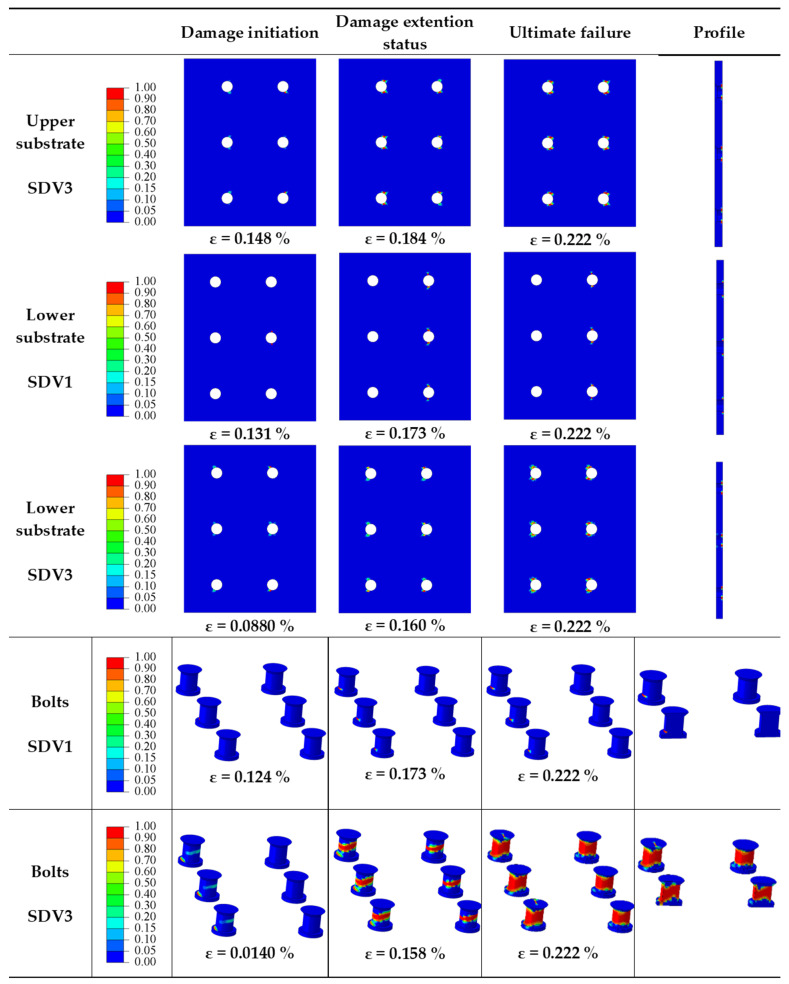
Damage processes in joint structures at *d_h_/t* = 1.7.

**Figure 15:**
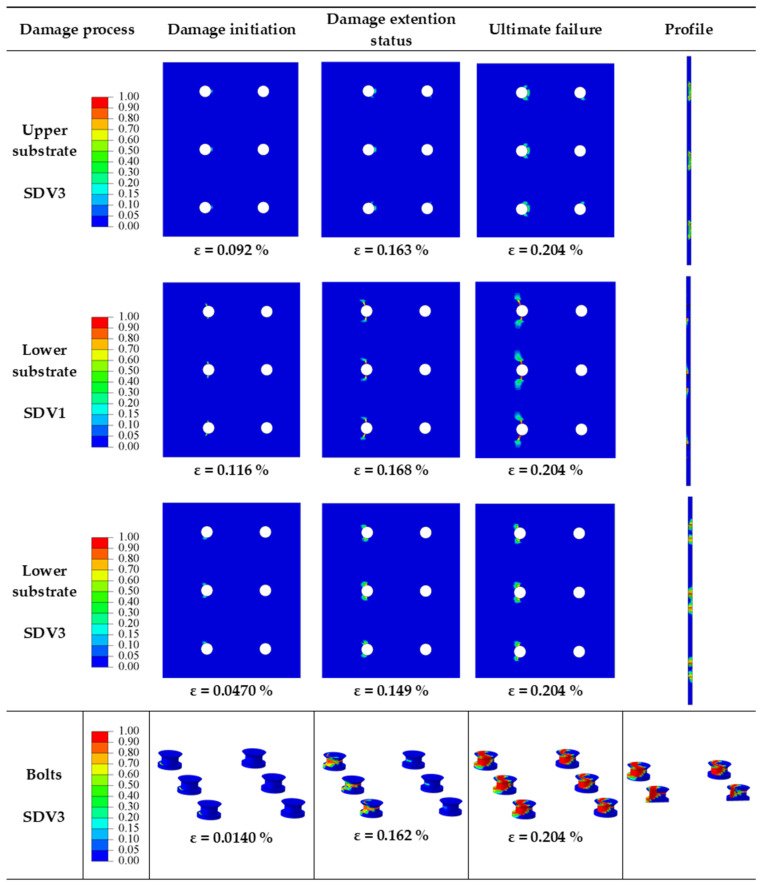
Damage processes in joint structures at *d_h_/t* = 3.3.

The objective of this section is to investigate the impact of *d_h_/t* on the mechanical behavior of multi-bolt, countersunk C/SiC composite joint structures. When *P/d_h_* = 5, *S/d_h_* = 5, *e/d_h_* = 3, *S_w_/d_h_* = 2.5, and a preload force of 2 kN, the effect of *d_h_/t* on the damage process of the joint structure is analyzed. The numerical model was used to analyze the effect of *d_h_/t* on the joint structure for *d_h_/t* of 1.7, 2, 2.5, and 3.3, respectively. These designs are numbered 11, 12, 4, and 13. Keeping the through hole diameter at 10 mm, the corresponding substrate thicknesses are 6 mm, 5 mm, 4 mm, and 3 mm. The damage process and damage pattern are similar for *d_h_/t* = 1.7 and *d_h_/t* = 2. The damage process for *d_h_/t* = 1.7 is shown in Figure 14 as an example. The damage process for *d_h_/t* = 2.5 is shown in Figure 6. The damage process for *d_h_/t* = 3.3 is shown in Figure 15.

From the diagram of the damage process at *d_h_/t* = 1.7 in Figure 14, the damage occurs in different forms in the upper substrate, lower substrate, and bolts. In the upper substrate, the warp and weft fiber show no damage, while the matrix damage (SDV3) occurs. In the upper substrate, matrix damage (SDV3) initially appears in the shear-out damage region at the top and bottom of each bolt hole, with damage extending in the shear-out damage region as the load gradually increases. The shear stress is the main cause of the compression failure of the matrix in the shear-out damage region. The warp fiber damage (SDV1) and matrix damage (SDV3) of the lower substrate appear. In the lower warp, the damage first appears in the tensile area of the bolt holes near the fixed-end row. As the load continues to increase, the damage extends to the area of tensile damage. When loaded to peak load, the damage does not continue to expand. The matrix damage in the lower substrate is similar to the upper substrate in terms of compression damage pattern. The warp fiber damage (SDV1) first appears at the root of the row of bolts near the fixed end, and as the load increases, the damage extends along the root in all directions. When loaded to the peak load, the warp fiber damage does not extend to other areas. It is clear that the warp fiber damage is due to compression stresses resulting in compression damage to the warp fiber. The matrix damage (SDV3) also appears first at the root of the row of bolts near the fixed end, while the damage also occurs in the middle part of the bolts. As the load continues to increase, the damage to the root of the bolt and the damage to the middle part of the bolt spreads around and connects, with the same damage occurring to the row of bolts near the loaded end. When loaded to the peak load, the bolt matrix damage is most severe in the middle of the bolt and extends to the interior of the bolt. From the results, it is known that the damage produced in the root region is due to compressive damage to the matrix caused by the compression stress and that in the middle region, it is mainly due to compression damage to the matrix caused by the shear stress.

The damage process and damage pattern of the lower substrate are the same as for *P/d_h_* = 2.5 in Figure 6. In particular, the damage area of the lower substrate from the warp fiber damage (SDV1) in the tensile damage region and in the shear-out damage region is enlarged. The reduction in substrate thickness reduces the number of warp fibers that resist the tensile load, resulting in quick damage to these fibers.

**Figure 16:**
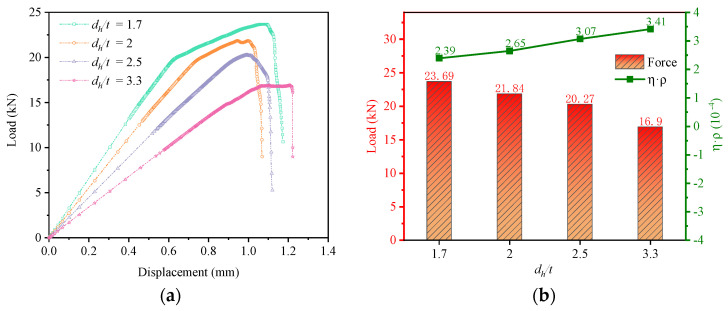
Comparison of results at different *d_h_/t*: (**a**) Load−displacement curves; (**b**) Load−bearing capacity and weight increment efficiency.

Figure 16a shows the load-displacement curves for different *d_h_/t*. As the substrate thickness increases, the initial stiffness of the joint structure increases significantly, and the strength of the joint structure improves, which indicates that increasing the substrate thickness will increase the load-bearing capacity of the structure. Combined with Figure 16b, increasing the substrate thickness will result in a reduction in the efficiency of the weight increment.

**Figure 17:**
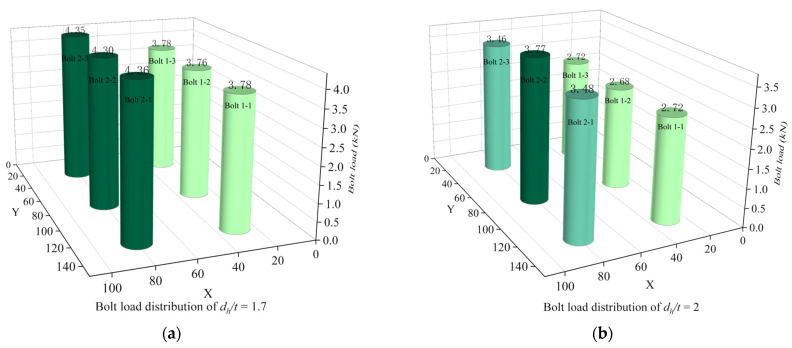

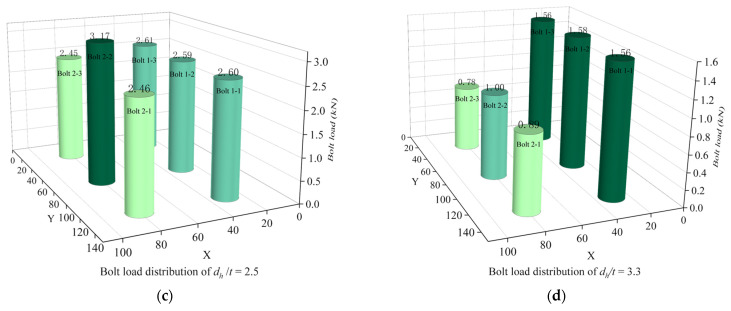
Bolt load distribution at different *d_h_/t*. (**a**) Bolt load distribution of *d_h_/t* = 1.7; (**b**) Bolt load distribution of *d_h_/t* = 2; (**c**) Bolt load distribution of *d_h_/t* = 2.5; (**d**) Bolt load distribution of *d_h_/t* = 3.3.

Figure 17a–d show the distribution of bolt load for different *d_h_/t*. In terms of the uniformity of the load-bearing capacity, the bolt load distribution for each row of bolts for the joint structure with *d_h_/t* = 1.7 is relatively uniform, and the difference in load-bearing capacity between the two rows of bolts is small.

Combining the load-bearing capacity, the weight increment efficiency, and the bolt load distribution, although the weight increment efficiency of the joint structure is highest at *d_h_/t* = 3.3, the joint structure at *d_h_/t* = 1.7 has the highest load-carrying capacity and the most uniform bolt load distribution. The best mechanical behavior of multi-bolt, countersunk C/SiC composite joint structures is achieved when *d_h_/t* = 1.7.

### 4.5. Effect of S_w_/d_h_ on Mechanical Behavior of Joint Structure

**Figure 18:**
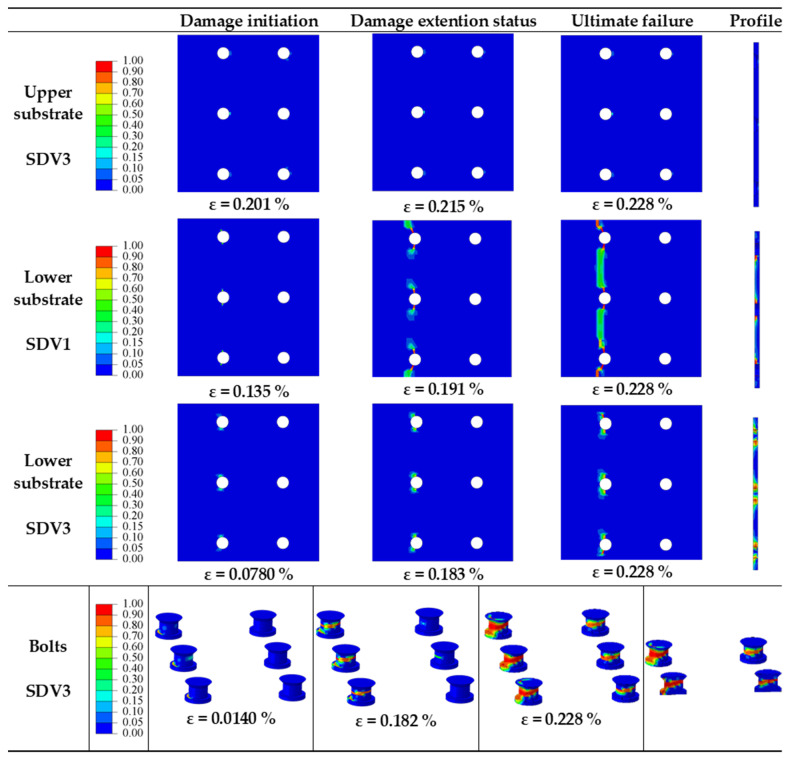
Damage processes in joint structures at *S_w_/d_h_* = 1.5.

The objective of this section is to investigate the impact of *S_w_/d_h_* on the mechanical behavior of multi-bolt, countersunk C/SiC composite joints. When *P/d_h_* = 5, *S/d_h_* = 5, *e/d_h_* = 3, *d_h_/t* = 2.5, and the preload force is 2 kN, the effect of *S_w_/d_h_* on the joint structure is analyzed. The *S_w_/d_h_* is 2.5, 1.5, 3.5, and 4.5 for the designs numbered 4, 14, 15, and 16, respectively. Analysis and comparison revealed that the damage process and damage pattern of the joint structure are similar for *S_w_/d_h_* of 2.5, 3.5, and 4.5, which can be referred to in Figure 6. The damage process and damage pattern changed when *S_w_/d_h_* is 1.5, and the damage process is shown in Figure 18.

The damage process diagram at *S_w_/d_h_* of 1.5 shows matrix damage (SDV3) is produced in the upper substrate, warp fiber damage (SDV1) and matrix damage (SDV3) occur in the lower substrate, and matrix damage occurs in the bolt matrix. The damage to the upper substrate occurs when the peak load is reached, and the damage is in the bearing damage region of the bolt holes in the row near the loading end with quite little area. The warp fiber damage (SDV1) of the lower substrate is initially in the net-tension damage region near the row of bolt holes at the fixed end. As the applied load increases, the damage extends along the net-tension damage region. At peak load, the damage has extended from the bolt holes near the edge of the substrate to the edge. The section shows that there is through damage at the bolt holes when the peak load is reached. The warp fibers are subjected to tensile stresses, resulting in damage in the net-tensile region. Due to the relatively minor distance between the substrate edges, the damage soon extends to the substrate edges, resulting in the structure losing its load-bearing capacity. The matrix damage (SDV3) to the base of the lower substrate first appeared in the shear area near the bolt holes in the fixed end of the row, and as the load continued to increase, the damage expanded into the shear area. The section shows that by the time the peak load is reached, the matrix damage has penetrated the entire bolt hole. The matrix damage (SDV3) to the bolt matrix first appears at the root of the bolt in the row near the fixed end. As the load continues to increase, due to the compression stress, damage starts to occur in the middle of the bolt, and this damage also occurs in the middle of the row of bolts near the loading end. When loaded to the peak load, the profile shows matrix damage through the entire bolt section, and the damage is more severe in the row of bolts near the fixed end. The damage to the root of the bolt is due to the compression of the matrix caused by the compression stress. The matrix damage in the middle of the bolt is mainly due to shear damage caused by shear and compression stresses.

**Figure 19:**
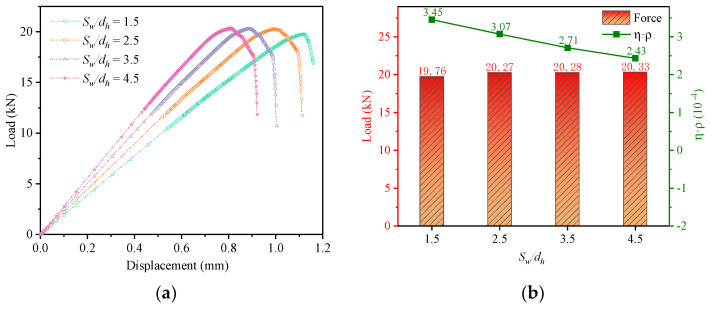
Comparison of results at different *S_w_/d_h_*: (**a**) Load−displacement curves; (**b**) Load−bearing capacity and weight increment efficiency.

Figure 19a shows the force-displacement curves for different *S_w_/d_h_*. As the edge spacing increases, the initial stiffness of the joint structure increases, but the strength of the structure remains the same. From Figure 19b, it can be seen that the efficiency of the weight increment of the structure decreases as *S_w_/d_h_* increases.

**Figure 20:**
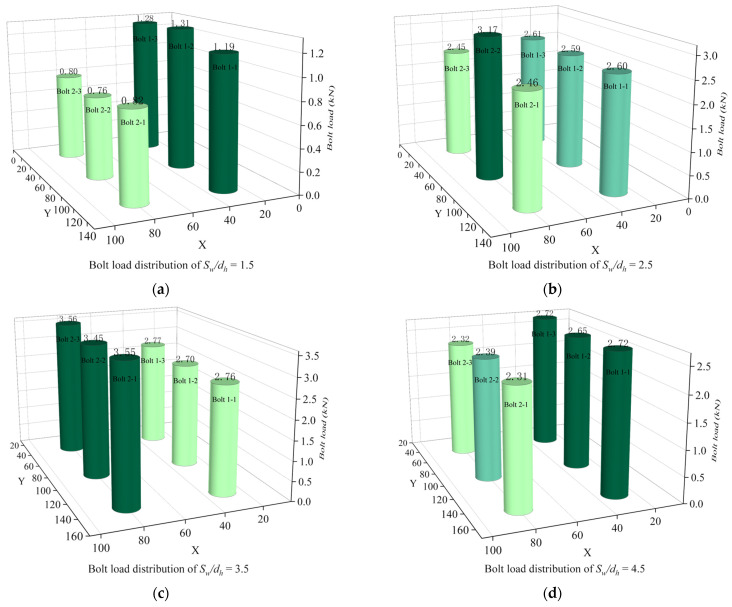
Bolt load distribution at different *S_w_/d_h_*. (**a**) Bolt load distribution of *S*_w_/*d_h_* = 1.5; (**b**) Bolt load distribution of *S*_w_/*d_h_* = 2.5; (**c**) Bolt load distribution of *S*_w_/*d_h_* = 3.5; (**d**) Bolt load distribution of *S*_w_/*d_h_* = 4.5.

Figure 20 shows the bolt load distribution for different *S_w_/d_h_*. In terms of uniformity of bolt load distribution, each row of bolts with *S_w_/d_h_* of 1.5 and 3.5 has a more uniform distribution of bolt load. *S_w_/d_h_* = 3.5 gives a smaller difference in bolt load distribution between the front and rear rows of bolts and a better performance.

Summarizing all the above analyses of the design parameters of the joint structure, we can get the following:

(1) The load capacity of the multi-bolt, countersunk C/SiC composite joint structure remains consistent as the ratio of bolt pitch to through hole diameter increases. The optimal ratio for this joint structure is 3 due to balanced bolt load distribution and efficient weight increment, while the damage pattern is primarily bolt matrix damage for ratios between 2 and 5.
(2) The load capacity of the multi-bolt, countersunk C/SiC composite joint structure increases with the ratio of bolt spacing to through hole diameter. The optimal ratio for this joint structure is 4, resulting in uniform bolt load distribution and efficient weight increment. The damage pattern shifts from warp tensile damage to lower substrate for ratios between 2 and 4 and to bolt matrix damage when the ratio reaches 5.
(3) The load capacity of the multi-bolt, countersunk C/SiC composite joint structure remains constant as the ratio of the distance between the free edge of the substrate to through hole diameter increases. The optimal ratio for this joint structure is 1.5, resulting in uniform bolt load distribution and maximum weight increment efficiency. The damage pattern is primarily bolt matrix damage for ratios between 1.5 and 3.
(4) Increasing the ratio of through hole diameter to specimen thickness decreases the load-carrying capacity of the multi-bolt, countersunk C/SiC composite joint structure. However, it increases the weight increment efficiency. The most uniform bolt load distribution is observed at a ratio of 1.7. The optimal performance of the joint structure is achieved at a ratio of 1.7, with a change in the damage pattern occurring at a ratio of 2. The predominant damage pattern is bolt matrix damage for ratios between 1.7 and 2, while warp damage to the lower substrate becomes dominant for ratios between 2.5 and 3.3.
(5) The load capacity of the multi-bolt, countersunk C/SiC composite joint structure remains constant as the ratio of the distance between the edge of the substrate to through hole diameter increases. The optimal ratio for this joint structure is 1.5, resulting in uniform bolt load distribution and reduced weight increment efficiency. The damage pattern shifts from warp damage to the lower substrate at a ratio of 1.5 to bolt substrate damage for ratios between 2.5 and 4.5.

## 5. Conclusions

In order to solve the problems of poor aerodynamic profile of the joint structure, mismatch of material thermal expansion coefficients, and a single evaluation index for the joint structure, this paper designs a multi-bolt, countersunk C/SiC composite joint structure. A 3D-Hashin progressive damage model is developed, along with a corresponding model solver code. The model’s validity is established through a comparison between simulation and experimental results. Three evaluation indexes were proposed, which are peak load, weight increment efficiency, and bolt load distribution, to evaluate the mechanical performance of multi-bolted, countersunk C/SiC composite joint structures. By employing the proposed model and evaluation index, 16 designs of multi-bolted, countersunk C/SiC composite joint structures were evaluated, and the impact of design parameters on their mechanical properties and damage patterns was analyzed. The main conclusions are:

(1) The optimal schemes derived from the three metrics of peak load, pin load distribution, and weight increment efficiency are a ratio of bolt pitch to through hole diameter of 3, a ratio of bolt spacing between columns to through hole diameter of 4, a ratio of distance between the free edge of the substrate to through hole diameter of 1.5, a ratio of through hole diameter to specimen thickness of 1.7, and a ratio of distance between the edge of the substrate to through hole diameter of 1.5, respectively.
(2) Peak load increases with increasing ratio of bolt spacing between columns to through hole diameter and the ratio of through hole diameter to specimen thickness. Changes in the ratio of bolt pitch to through hole diameter, the ratio of the distance between the free edge of the substrate to through hole diameter, and the ratio of the distance between the edge of the substrate to through hole diameter would not affect the peak load.
(3) In multi-bolt joint structures with different design parameters, matrix damage (SDV3) occurs in the bolts. Warp damage (SDV1) occurs in the net-tension damage region of the lower substrate when the ratio of bolt spacing to through hole diameter is not greater than 4 or when the ratio of distance between the edge of the substrate to through hole diameter is not greater than 1.5.

## Figures and Tables

**Table 1:** Material properties [20].

Modulus (Gpa)	*E*_1_ = *E*_2_	*E*_3_	*G*_12_	*G*_13_ = *G*_23_	Poisson Ratio	*ν*_12_	*ν*_13_ = *ν*_23_
	120	60	44.4	24		0.25	0.35
Strength (Mpa)	*X*_T_ = *Y*_T_	*X*_C_ = *Y*_C_	*Z*_T_	*Z*_C_	*S*_12_	*S*_13_ = *S*_23_	
	238.91	409.40	60.31	120.19	114.53	34.68	

**Table 2:** Design parameters.

No.	*P/d_h_*	*S/d_h_*	*e/d_h_*	*d_h_/t*	*S_w_/d_h_*	*P* (mm)	*S* (mm)	*e* (mm)	*S_w_* (mm)	*W × t × L* (mm^3^)
1	2	5	3	2.5	2.5	20	50	30	25	150 × 4 × 300
2	3	5	3	2.5	2.5	30	50	30	25	150 × 4 × 300
3	4	5	3	2.5	2.5	40	50	30	25	150 × 4 × 300
4	5	5	3	2.5	2.5	50	50	30	25	150 × 4 × 300
5	5	2	3	2.5	2.5	50	20	30	25	90 × 4 × 300
6	5	3	3	2.5	2.5	50	30	30	25	110 × 4 × 300
7	5	4	3	2.5	2.5	50	40	30	25	130 × 4 × 300
8	5	5	1.5	2.5	2.5	50	50	15	25	150 × 4 × 300
9	5	5	2	2.5	2.5	50	50	20	25	150 × 4 × 300
10	5	5	2.5	2.5	2.5	50	50	25	25	150 × 4 × 300
11	5	5	3	1.7	2.5	50	50	30	25	150 × 6 × 300
12	5	5	3	2	2.5	50	50	30	25	150 × 5 × 300
13	5	5	3	3.3	2.5	50	50	30	25	150 × 3 × 300
14	5	5	3	2.5	1.5	50	50	30	15	130 × 4 × 300
15	5	5	3	2.5	3.5	50	50	30	35	170 × 4 × 300
16	5	5	3	2.5	4.5	50	50	30	45	190 × 4 × 300

## Data Availability

Not applicable.
